# Numerical Modeling of Transient Flow Characteristics on the Top Surface of a Steel Slab Continuous Casting Strand Using a Large Eddy Simulation Combined with Volume of Fluid Model

**DOI:** 10.3390/ma16165665

**Published:** 2023-08-17

**Authors:** Yushi Tian, Haichen Zhou, Guobin Wang, Lijun Xu, Shengtao Qiu, Rong Zhu

**Affiliations:** 1 School of Metallurgical and Ecological Engineering, University of Science and Technology Beijing (USTB), Beijing 100083, China; yushi_tian@tom.com (Y.T.);; 2 National Engineering Research Center of Continuous Casting Technology, Central Iron and Steel Research Institute, Beijing 100081, China; 3 Research Institute of Technology, Shougang Group Co., Ltd., Beijing 100043, China

**Keywords:** transient flow characteristics, continuous casting mold, top surface level profile, instantaneous distribution of vortex, instantaneous level fluctuation

## Abstract

In the current study, the transient flow characteristics on the top surface of a steel slab continuous casting strand were numerically investigated using a large eddy simulation combined with volume of fluid (LES + VOF) model. The validation of numerical simulation was verified via nail board measurement in the industrial continuous casting mold. The effects of casting speed on the top surface level profile and the instantaneous distribution of vortex were discussed. The level variation profile migrated after a period of time, moving from one side of the wide face of the mold to the other. The wave height and transient variation degree of the standing wave increased with an increase in the casting speed. The region near the SEN was more likely to promote the formation of vortices. The vortex generation became easier when the vorticity peaks were concentrated on the outer edge of the low-speed confluence area near the submerged entry nozzle. In addition, the effect of surface velocity on the instantaneous level fluctuation was analyzed. The frequency of level fluctuations was highest at 3~4 mm, and the high-frequency range of velocity fluctuation was 20~60 mm/s at 0.9 m/min casting speed for a 1500 mm × 200 mm caster section. The linear relationship between the level fluctuation and surface velocity magnitude was obtained. The present work aimed at evaluating the dynamic problem of the standing wave at the liquid powder–molten steel interface on the top surface of the mold, which is helpful in optimizing the casting parameters for regular casting practice and improving the quality of the steel slabs.

## 1. Introduction

The flow of molten steel on the top surface of a steel slab continuous casting (CC) mold is complex, with strong heat transfer, mass transfer, and transient variation [1]. In addition, the molten steel flow on the top surface is covered with a liquid slag layer of mold powder, which can be regarded as a multi-phase and multi-physical field coupling flow of high-temperature stratified melt. The transient flow characteristics of the slag–steel interface, such as strong surface velocity, top surface level profile (TSLP) [2,3], standing wave height [4,5], instantaneous distribution of vortex [6,7,8], and instantaneous level fluctuation [9,10], directly affect the occurrence of slag entrainment and the quality of the steel slabs [11]. Therefore, understanding the transient variation characteristics of molten steel flow on the top surface of a mold is essential and indispensable for industrial engineers.

During the CC process, there are obvious peaks and troughs on the top surface of the mold on both sides of the submerged entry nozzle (SEN) [5]. Peaks and troughs are approximately symmetrical in time with the SEN as the center, and this surface waveform is the standing wave (SW) [12,13]. In addition, there are fluctuations and disturbances in the wave surface under the influence of different factors [5,13,14]. Under the double-roll flow (DRF) pattern [15,16], the trough is at the 1/4 mold width, where the liquid level is the lowest [17,18]. The level fluctuation of the top surface of the mold is caused by the superposition effect of the SW formed by the slag–steel interface and the transient disturbance of the liquid level, which is dominated by the casting speed, argon flow rate, submergence depth of the SEN, geometric structure of the SEN, width–thickness ratio of casting section, melt physical properties, etc. [19]. The level fluctuation of the top surface of the mold is considered to be the result of the combined action of the impact of the molten steel flow on the liquid level and the bulging of the steel slab. Recent studies on the level fluctuation of the top surface of the CC mold are summarized in Table 1. Most works have studied causes of level fluctuation on the top surface of the CC mold, including the impact of molten steel flow, bias flow, argon blowing, static pressure change in molten steel, disturbance of the stopper rod, fluid flow pattern in the mold, etc. However, the transient flow characteristics on the top surface were rarely studied, especially the dynamic problem of the SW and the relationship between level fluctuation and surface velocity magnitude under different casting speeds of the CC mold. The analysis of the transient flow characteristics on the top surface of the CC mold is helpful to optimize the casting parameters for regular casting practice.

In the current work, the transient flow characteristics on the top surface of the mold at different casting speeds were investigated using a large eddy simulation combined with volume of fluid (LES + VOF) model. The nail board measurement was adopted to validate the accuracy of the numerical method. Then, the effects of casting speed on the TSLP and instantaneous distribution of vortex were discussed. In addition, the effect of surface flow velocity on instantaneous level fluctuation was studied. This work was used to qualitatively evaluate the dynamic problem of the SW at the liquid slag–molten steel interface on the top surface of the mold as well as the relationships between the relevant characteristic parameters, which is helpful in optimizing casting parameters and improving the product quality.

## 2. Computational Models

### 2.1. Assumptions

In the mathematical model, assumptions are specified as follows:

1. The molten steel is treated as incompressible Newtonian fluids, and the process of solidification and heat transfer in the mold are ignored;
2. The physical properties of molten steel and mold powder are set as constants;
3. The computational domain extends from the mold to the bending zone, and its direction is simplified to vertical downward;
4. The molten steel flow at the outlet of calculation domain is assumed to be fully developed turbulence.

### 2.2. Continuity Equations

For the mixed melt composed of molten steel and mold powder, the continuity and momentum equations are written as follows:

Continuity equation:

(1) $$\frac{\partial \rho }{\partial t}+\frac{\partial }{\partial x_{i}}(\rho u_{i})=0$$

where *ρ* is density of the mixed melt in kg/m^3^, *t* is time in s, and *u_i_* is the velocity in m/s, respectively.

The mixed melt density is defined as:

(2) $$\rho =f_{s}\rho_{s}+f_{f}\rho_{f}$$

where *f* is the volume fraction, and the subscripts *s* and *f* denote the mold powder phase and molten steel phase, respectively.

Momentum equation:

(3) $$\frac{\partial (\rho u_{i})}{\partial t}+\frac{\partial \left(\rho u_{i}u_{j}\right)}{\partial x_{j}}=-\frac{\partial P}{\partial x_{i}}+\frac{\partial }{\partial x_{j}}\left[\left(\mu +\mu_{t}\right)\left(\frac{\partial u_{i}}{\partial x_{j}}+\frac{\partial u_{j}}{\partial x_{i}}\right)\right]+\rho g_{i}+S_{\sigma_{i}}$$

where *P* is the pressure in Pa, *μ* is the dynamic viscosity in Pa·s, *μ_t_* is the turbulent viscosity in Pa·s, *g* is the gravity acceleration in m/s^2^, and *S_σi_* is the surface tension force in N/m^3^, respectively.

The surface tension force is defined as [31]:

(4) $$S_{\sigma i}=\frac{\partial }{\partial x_{i}}\left(\frac{\sigma_{sf}\rho \kappa f_{s}}{\frac{1}{2}\left(\rho_{s}+\rho_{f}\right)}\right)$$

where *σ_sf_* is the surface tension coefficient in N/m, and *κ* is the curvature in m^−2^.

The curvature is calculated as:

(5) $$\kappa =\nabla \cdot \hat{n}$$

where $\hat{n}$ is the surface normal, $\hat{n}=\frac{n}{\left|n\right|}$, $n=\nabla f_{s}$.

### 2.3. LES Model

For the LES model, the large eddies are resolved directly, and the small eddies are modeled, respectively. The turbulent viscosity in the Smagorinsky–Lilly subgrid-scale model is calculated as [32]:

(6) $$\mu_{t}=\rho L_{s}^{2}\left|\bar{S}\right|$$

where *L_s_* is the mixing length for subgrid-scales in m, and *S* is the average deformation rate tensor in s^−1^.

The mixing length for subgrid-scales and the average deformation rate tensor are calculated as:

(7) $$L_{s}=\min\left(\kappa d,C_{S}V^{\frac{1}{3}}\right)$$

(8) $$\left|\bar{S}\right|=\sqrt{2{\bar{S}}_{ij}{\bar{S}}_{ij}}$$

where *κ* is the von Kármán constant, *d* is the distance to the closest wall in m, *C_S_* is the Smagorinsky constant and equals to 0.1, and *V* is the volume of the computational cell in m^3^.

### 2.4. Volume of Fluid Model

The interface between the steel and slag phase is tracked and calculated using the VOF model [33]. This method of the tracing inter-phase boundary was achieved by solving one set of momentum equations for the mixture of phases, and one equation for the volume fraction of a fluid.

(9) $$\frac{\partial \left(f_{f}\rho_{f}\right)}{\partial t}+\frac{\partial }{\partial x_{i}}(f_{f}\rho_{f}u_{i})=0$$

The volume fraction of the slag and molten steel phase is given by

(10) $$f_{s}+f_{f}=1$$

where *f_s_* is the volume fraction of mold powder phase, and *f_f_* is the volume fraction of molten steel phase, respectively.

### 2.5. Numerical Details

**Figure 1:**
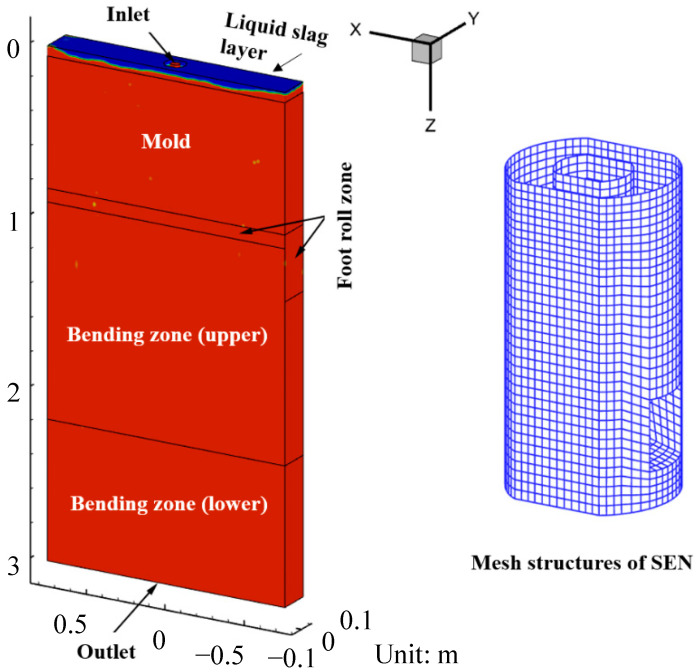
Schematic of the calculation domain and mesh.

In order to investigate the transient flow characteristics on the top surface of the mold, a fluid flow mathematical model for an actual steel slab CC strand was established. A full-size scale model was modeled based on the actual mold. The length of the calculation domain was 3 m to eliminate the influence of the molten steel flow circulation zone in the CC process. The total number of structured mesh cells for the large eddy simulation was approximate 1.4 million, and the fine graded mesh was adopted near the wall surface. Figure 1 shows the schematic of the calculation domain and mesh. The inlet boundary condition was velocity inlet based on the mass conservation. The outlet boundary condition was pressure outlet, and the normal gradients of all dependent variables were set to zero. The top surface of the mold was supposed to be flat and insulated using a free-slip condition. Other dimensions and physics parameters used in the numerical simulation are given in Table 2. Simulations were carried out on the software platform of CFD code ANSYS-Fluent 2021. The pressure–velocity coupling was solved using the SIMPLE algorithm. The discretization of the momentum equation was solved using the Bounded Central Differencing scheme. The convergence criteria was set to 1 × 10^−4^ for the residuals of all transport equations. Time step was set as 5 × 10^−4^ s. The total calculation time was approximately 200 s to obtain the quasi-steady state.

## 3. Nail Board Measurements

**Figure 2:**
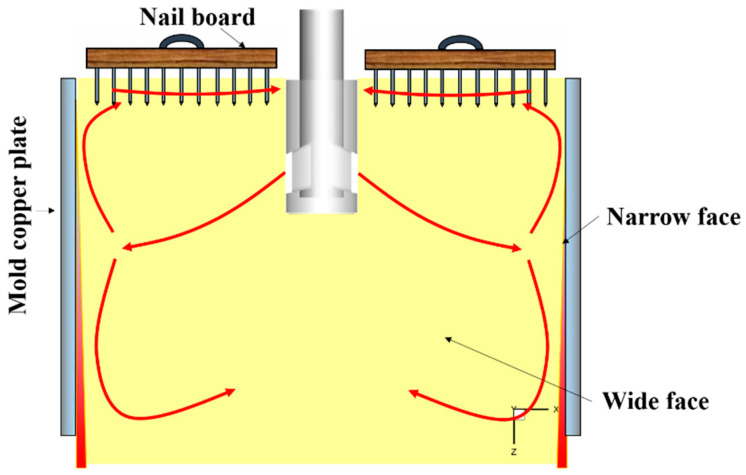
Schematic of the nail board measurement.

In order to validate the accuracy of the numerical method, the nail board measurement was adopted to measure the surface velocity of the molten steel in the industrial CC mold [34,35,36]. Figure 2 shows the schematic of the nail board measurement. In the experiment, the nail board was inserted into the molten steel vertically through the mold powder layer, and it stayed for 3~5 s before being pulled out. The depth of the steel nail inserted into the molten steel was in the range of 40~60 mm. The nail board measurement was carried out simultaneously on both sides of the SEN. The surface velocity of molten steel can be calculated by measuring the height difference between the incoming direction and the de-flow direction of the molten steel, and the diameter of the solidified lump at the lowest point of the de-flow direction. The calculation equation of the surface velocity is as follows [25]:

(11) $$V_{s}=0.624\;\varphi_{lump}^{-0.696}\;h_{lump}^{0.567}$$

where *V_s_* is the steel surface velocity in m/s, *φ_lump_* is the lump diameter in mm, and *h_lump_* is the difference in lump height in mm.

Casting parameters in industrial trials are the same as in the numerical simulation and given in Table 2. The compositions of the steel are listed in Table 3. The compositions of the original mold powder are given in Table 4.

## 4. Results and Discussion

### 4.1. Numerical Simulation Validation

**Figure 3:**
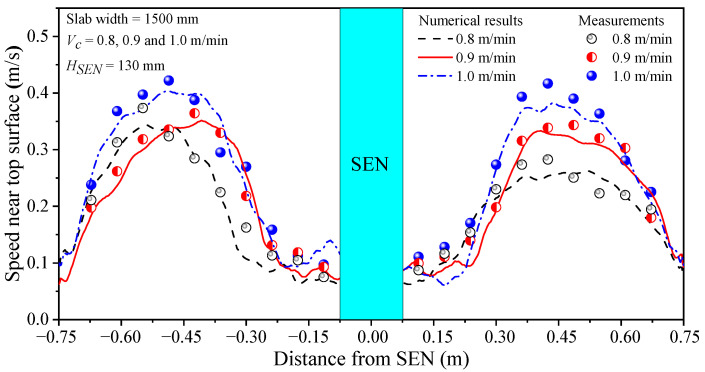
Comparison of calculated and measured speed of molten steel near the surface.

In order to verify the accuracy of the mathematical model, the measured and calculated speed of molten steel along the thickness center line of the top surface of the mold are compared, as shown in Figure 3. The numerical simulation results were in good agreement with the measured values. Therefore, the fluid flow mathematical model established in the current study can be used to predict the molten steel flow in the mold.

### 4.2. Effect of Casting Speed on Top Surface Level Profile

**Figure 4:**
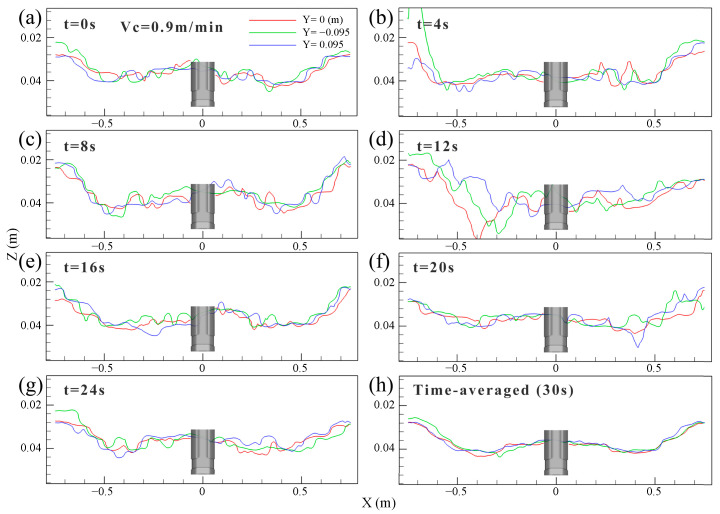
Transient and time-averaged distribution of top surface level profile of the mold. (**a**) t = 0 s. (**b**) t = 4 s. (**c**) t = 8 s. (**d**) t = 12 s. (**e**) t = 16 s. (**f**) t = 20 s. (**g**) t = 24 s. (**h**) t = 30 s.

Figure 4 shows the transient and time-averaged distribution of top surface level profile at the interface between the molten steel and slag layer in the mold within 30 s and at 0.9 m/min casting speed. The TSLP was taken from the slag phase volume fraction of 0.9, and its standing wave profile was characterized by the three characteristic lines at different positions. The red line was the center line of the liquid surface (Y = 0 m ), the green line was located at 0.005 m (Y = −0.095 m ) from the fixed face, and the blue line was located at 0.005 m (Y = 0.095 m ) from the loose face of the mold, respectively. The level profile of the mold showed significant level fluctuation at different times, as shown in Figure 4a–g. The characteristic line shows a tortuous and uneven shape, indicating the liquid level changed greatly, as shown in Figure 4c–d, which made it easy to induce the slag entrainment. However, this sudden variation in the shape of the level profile migrated after a period of time, moving from one wide face of the mold to the other side. Although the level fluctuation degree at different positions varied, it did not significantly change the position of the peak and the trough of the TSLP. As shown in Figure 4h, the wave peak of the level profile was near the SEN and the narrow face, while the trough was close to the 1/4 wide face, which was generally symmetrical with the SEN as the center. The above results showed that the instantaneous top level profile continues to oscillate periodically, although the time-averaged velocity distribution was in good symmetricity.

**Figure 5:**
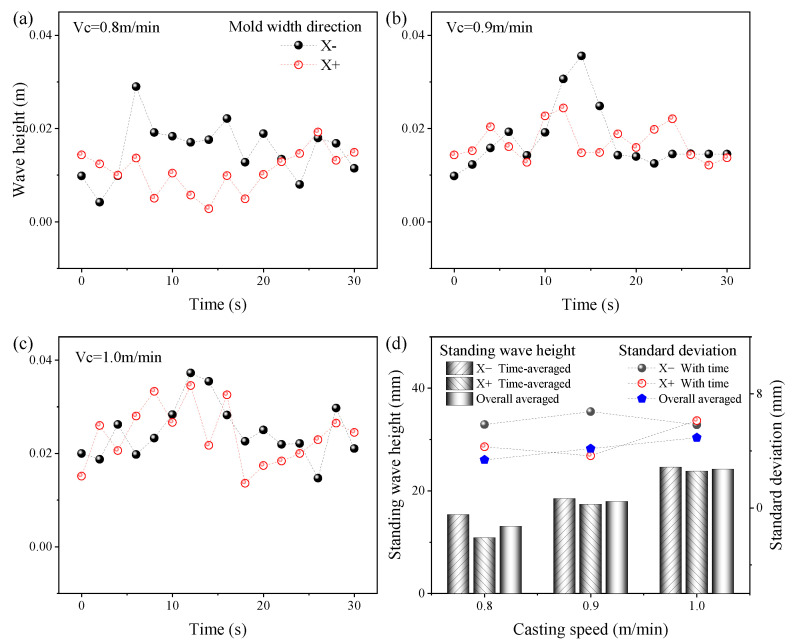
Variation in standing wave height with time at different casting speeds. (**a**) *V_c_* = 0.8 m/min; (**b**) *V_c_* = 0.9 m/min; (**c**) *V_c_* = 1.0 m/min; (**d**) Standing wave height and standard deviation.

The variation in standing wave height with time at different casting speeds is shown in Figure 5. The symbols X− and X+ denote the negative and positive directions along the mold width with the SEN as the center, respectively. From Figure 5a–c, the height of the SW was transient, but the transient degree of the top surface wave height on both sides of the SEN along the mold width was not synchronized. The magnitude of the standing wave height and the degree of transient expressed by the standard deviation at 30 s time-averaged condition are shown in Figure 5d. The time-averaged wave height and the transient degree of the SW increased with the increase in the casting speed. The increasing tendency of the SW was explained by the following mechanism. With the increase in the casting speed, a shorter time was needed for the molten steel moved from the SEN to the narrow face, which led to an increase in the momentum of upward movement of molten steel after impact on the narrow face. The increase in the height amplitude of the SW was attributed to an increase in the casting speed. At 0.8 m/min casting speed, the SW height on both sides of the mold width was quite different. This difference gradually decreased as the casting speed increased.

### 4.3. Effect of Casting Speed on Vortex Distribution

**Figure 6:**
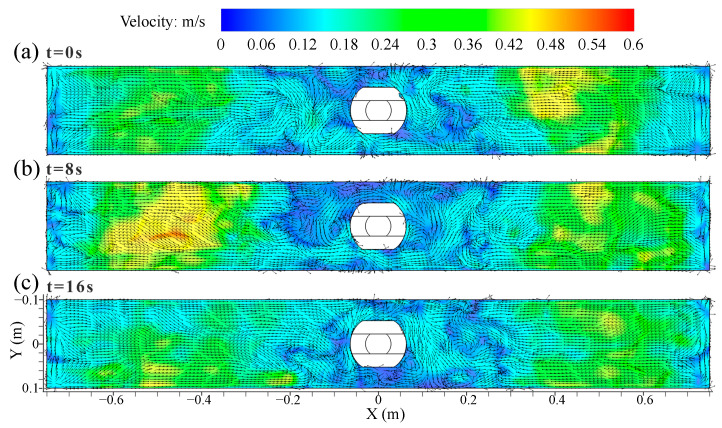
Transient velocity and vortex distribution on the top surface of the mold at casting speed of 0.9 m/min. (**a**) t = 0 s. (**b**) t = 8 s. (**c**) t = 16 s.

Figure 6 illustrates the transient velocity and vortex distribution on the top surface of the mold at 0.9 m/min casting speed. The surface velocity showed a clear asymmetric distribution under transient conditions. The velocity had a significant peak, and the value of the peak varied with the casting time. The peak value of the surface velocity appeared on the right side of the SEN at t = 0 s and on the left side of the SEN at t = 8 s along the mold width, as shown in Figure 6a,b, respectively. In Figure 6c, the peak value decreased, and the velocity distribution tended to be moderate at t = 16 s.

**Figure 7:**
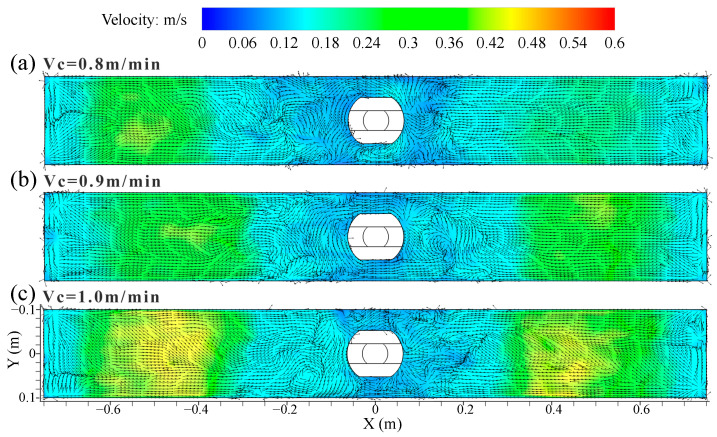
Distribution of time-averaged (30 s) velocity and vortex distribution on the top surface of the mold at different casting speeds. (**a**) *V_c_* = 0.8 m/min; (**b**) *V_c_* = 0.9 m/min; (**c**) *V_c_* = 1.0 m/min.

Figure 7 shows the distribution of time-averaged velocity and vortex distribution on the top surface of the mold at different casting speeds. The surface velocity was symmetrical with the SEN as the center, and it had the largest value at the 1/4 mold width, while the velocity near the SEN and the narrow face was smaller. The steel flow from the narrow face converged near the vicinity of the SEN under the DRF pattern, forming a low-speed confluence zone. There were many vortices of different sizes and shapes in the low-speed confluence area, and their number and position varied with the casting time. The region near the SEN was more likely to promote the formation of vortices. The meniscus vortices was also reported to largely occur near the SEN and it was moved far from the SEN [6]. These structures resembled the vortex shedding around a circular cylinder [7]. The vortexing flow on the top surface of the mold was an important factor for the slag entrainment [37].

**Figure 8:**
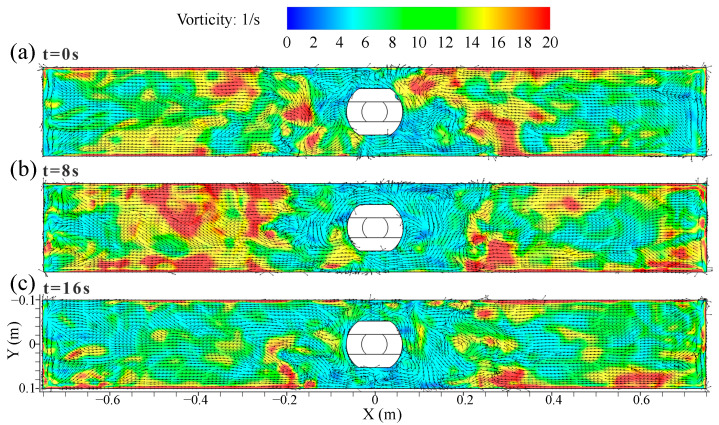
Distribution of transient vorticity and vortex on the top surface of the mold at casting speed of 0.9 m/min. (**a**) t = 0 s. (**b**) t = 8 s. (**c**) t = 16 s.

Figure 8 shows the distribution of the transient vorticity and vortex on the top surface of the mold at casting speed of 0.9 m/min. In Figure 8a–c, the vorticity had a large peak value at a certain time. When these peaks were concentrated on the outer edge of the low-speed confluence area near the SEN, it was beneficial to promote the formation of the vortex. The asymmetric vorticity distribution on the top surface of the mold was achieved via the effect of the turbulent instability.

**Figure 9:**
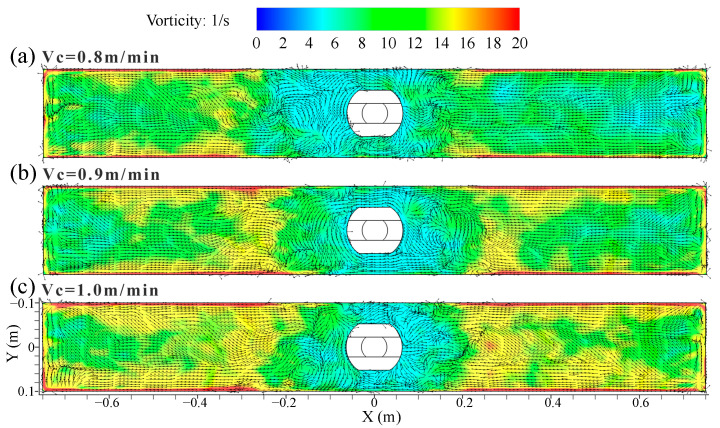
Distribution of time-averaged vorticity and vortex on the top surface of the mold at different casting speeds. (**a**) *V_c_* = 0.8 m/min; (**b**) *V_c_* = 0.9 m/min; (**c**) *V_c_* = 1.0 m/min.

Figure 9 illustrates the distribution of time-averaged vorticity and vortex on the top surface of the mold at different casting speeds. The vorticity was mainly distributed on the edge of the low-speed confluence area of the SEN towards the narrow face, and there was also a concentrated distribution of vorticity on the circumferential edge of the mold at an average time of 30 s. The reason for this phenomenon was that vortices were made near the border of the two streams approaching from the opposite sides.

**Figure 10:**
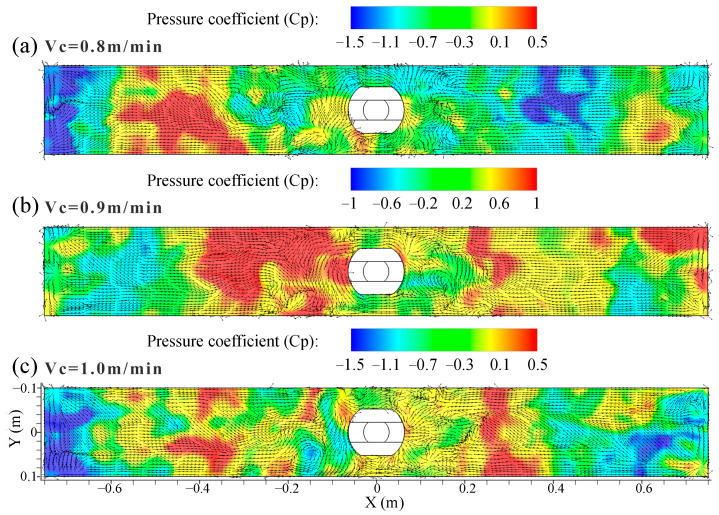
Distribution of time-averaged pressure coefficient on the top surface of the mold at different casting speeds. (**a**) *V_c_* = 0.8 m/min; (**b**) *V_c_* = 0.9 m/min; (**c**) *V_c_* = 1.0 m/min.

The distribution of time-averaged pressure coefficient on the top surface of the mold at different casting speeds is shown in Figure 10. The relative pressure of the top surface is expressed by the pressure coefficient, *C_p_*, and the equation is given by

(12) $$C_{p}=\frac{P_{x}-P_{0}}{P_{d}}$$

where *P_x_* is the local static pressure of top surface in Pa, *P*_0_ is the reference static pressure in Pa, and *P_d_* is the dynamic pressure in Pa.

The dynamic pressure is calculated as:

(13) $$P_{d}=\frac{\rho \cdot U_{h}^{2}}{2}$$

where *ρ* is the density of the mixed melt in kg/m^3^, and $U_{h}^{2}$ is the velocity of the mixed melt in m/s, respectively.

From Figure 10a–c, there is a low pressure zone near the narrow face of the mold, and the pressure in the low-speed confluence zone close to the SEN is higher than that near the narrow face. The pressure in the vortex center was obviously lower, which was more obvious on the left side of the SEN along the mold width at 1.0 m/min casting speed. Overall, the area where the relative pressure of the top surface was low can appear near the narrow face or close to the 1/4 mold width.

### 4.4. Effect of Surface Flow Velocity on Instantaneous Level Fluctuation

**Figure 11:**
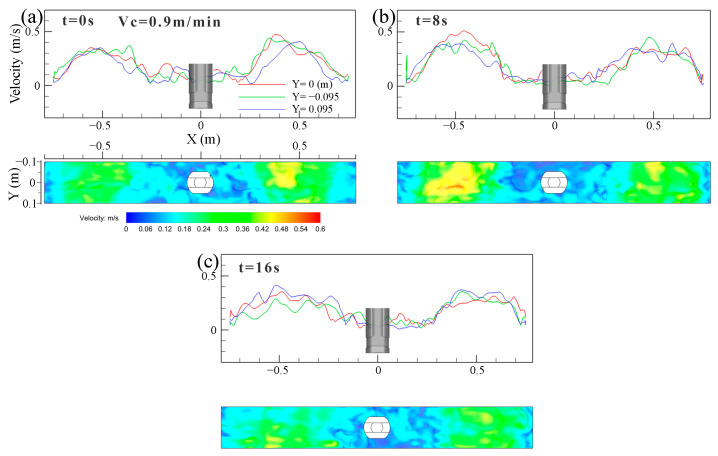
Transient velocity distribution of molten steel near the top surface of the mold at casting speed of 0.9 m/min. (**a**) t = 0 s. (**b**) t = 8 s. (**c**) t = 16 s.

The transient velocity of molten steel near the top surface of the mold at casting speed of 0.9 m/min is shown in Figure 11. In Figure 11a–c, the transient velocity of the molten steel near the top surface shows a saw-tooth level fluctuation, which changes with the casting time, and the symmetry of the top surface on both sides of the SEN disappears. The results shown that the fluctuation of the transient surface velocity on both sides of the SEN was disordered and violent.

**Figure 12:**
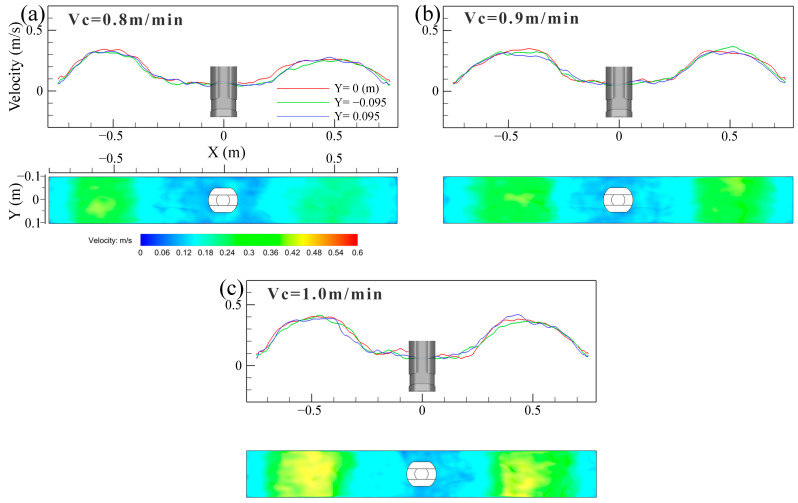
Distribution of time-averaged (30 s) velocity of molten steel near the top surface of the mold at different casting speeds. (**a**) *V_c_* = 0.8 m/min; (**b**) *V_c_* = 0.9 m/min; (**c**) *V_c_* = 1.0 m/min.

Figure 12 illustrates the distribution of time-averaged (30 s) velocity of the molten steel near the top surface of the mold at different casting speeds. The time-averaged velocity of molten steel distribution on both sides of the SEN is approximately symmetrical. The velocity was high at the 1/4 mold width, while it was low close to the narrow face and the SEN. As a whole, the surface velocity was derived from the impinged effect of the upper roll flow. If the casting speed was large, the upper roll flow had a larger momentum and velocity, and a larger impact on the meniscus resulted in a larger velocity.

**Figure 13:**
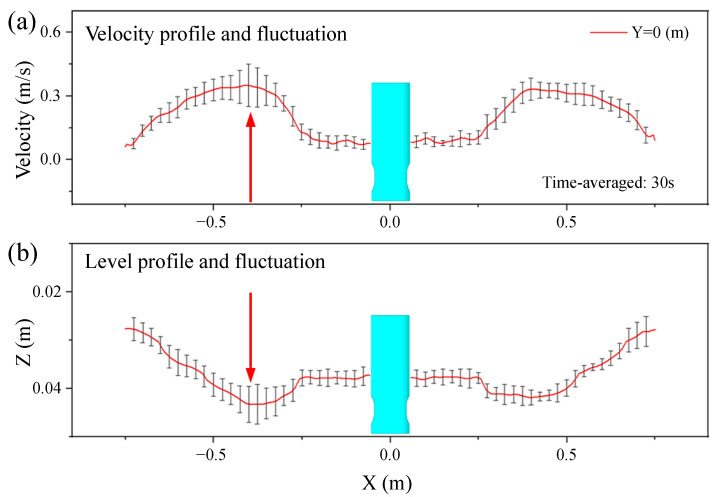
Distribution of velocity profile, level profile, and fluctuation on the top surface along the mold thickness center. (**a**) Velocity profile and fluctuation; (**b**) Level profile and fluctuation.

Figure 13 shows the distribution of velocity profile, level profile, and level fluctuation on the top surface along the mold thickness center at 0.9 m/min casting speed. The distribution of the centerline velocity and the corresponding level fluctuation of the top surface are shown in Figure 13a. The velocity fluctuation was larger at the 1/4 mold width, and it was smaller at the low speed zone near the narrow face and the SEN. The strong surface velocity and its fluctuation made it easy to cause the slag entrainment [38,39]. The distribution of the centerline level profile and the corresponding fluctuation of the top surface is shown in Figure 13b. The top surface level was the largest at the 1/4 mold width, which was consistent with the change trend of the velocity fluctuation. The liquid level fluctuation near the narrow surface was larger than that near the SEN.

**Figure 14:**
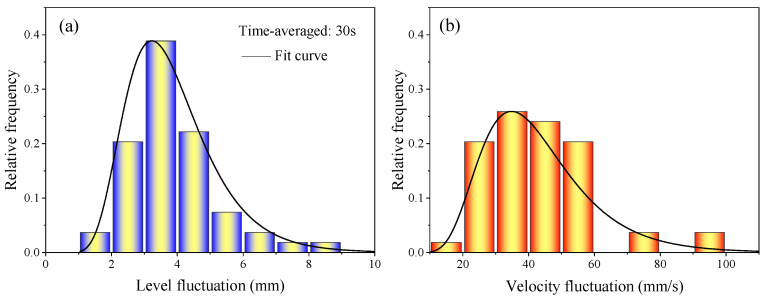
Relative frequency distribution of liquid level fluctuation and velocity fluctuation magnitude on the top surface of the mold. (**a**) Liquid level fluctuation; (**b**) velocity fluctuation.

The relative frequency distribution of the liquid level fluctuation and the velocity fluctuation magnitude on the top surface of the mold at 0.9 m/min casting speed is shown in Figure 14. The frequency of the level fluctuation at 2~6 mm was higher, and the level fluctuation at 3~4 mm was the highest frequency, which occupied the main position, as shown in Figure 14a. The high frequency range of velocity fluctuations was 20~60 mm/s, of which 30~40 mm/s occupied the main position, as shown in Figure 14b.

**Figure 15:**
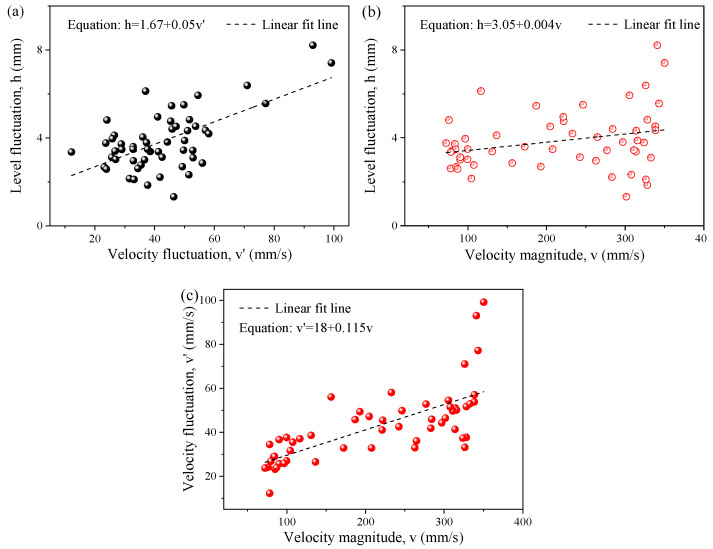
Relationship between the surface velocity, surface velocity fluctuation, and liquid level fluctuation. (**a**) Velocity fluctuation and level fluctuation; (**b**) velocity magnitude and level fluctuation; (**c**) velocity magnitude and velocity fluctuation.

Figure 15 shows the relationship between the surface velocity, surface velocity fluctuation, and liquid level fluctuation. The level fluctuation increased with an increase in the velocity fluctuation, as shown in Figure 15a, and the velocity fluctuation also increased with the increase in velocity magnitude, as shown in Figure 15c, which indicated that there was a positive correlation between the level fluctuation and the surface velocity magnitude, as shown in Figure 15b. Therefore, the level fluctuation can be controlled by adjusting the surface velocity magnitude. The greater the surface velocity, the stronger the trend of the shearing slag entrainment. The fitting relationship between the level fluctuation and the surface velocity magnitude was:

(14) h=0.004v+3.05

where *h* is the magnitude of the level fluctuation in mm, and *v* is the surface velocity magnitude in mm/s, respectively.

**Figure 16:**
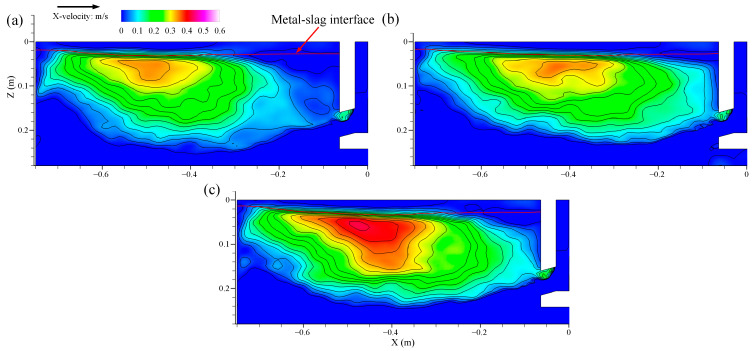
Comparison of the velocity gradient at the X-axis of slag–steel interface at different casting speeds. (**a**) *V_c_* = 0.8 m/min; (**b**) *V_c_* = 0.9 m/min; (**c**) *V_c_* = 1.0 m/min.

Figure 16 shows the comparison of the velocity gradient at the X-axis of the slag–steel interface at different casting speeds. The horizontal velocity along the mold width increased with the increase in the casting speed. The magnitude of the surface velocity in the X direction directly affected the magnitude of the shear force. The horizontal velocity gradient at the 1/4 mold width was larger, indicating that the shear force was stronger, which meant it was easier to induce the slag entrainment [27].

## 5. Conclusions

In the current study, the transient flow characteristics on the top surface of a steel slab CC strand was studied using a LES + VOF model. The simulation results of the molten steel speed along the thickness center line of the top surface of the mold matched well with the nail board measurements. The effects of casting speeds on TSLP and instantaneous distribution of vortex were discussed. In addition, the effects of surface flow velocity on instantaneous level fluctuations were analyzed, and quantitative research has been conducted on the relationship between the velocity of the surface flow and level fluctuations of the slab mold. This work is helpful in optimizing the casting parameters for regular casting practices in a steel plant, contributing to the production of defect-free continuous cast slabs, and improving the production efficiency. Our conclusions are summarized as follows:

1. The level variation profile migrated after a period of time, moving from one width side of the mold to the other side. Under the DRF pattern, with the increase in the casting speed, the SW height of the top surface increased, and the transient fluctuation degree of the wave height increased.
2. The vortex on the top surface of the mold was easy to generate in the low-speed confluence area near the SEN. The velocity, vorticity, and relative pressure of the vortex center were small, while the velocity and vorticity of the edge were large. When the vorticity peak was concentrated on the outer edge of the low-speed confluence zone close to the SEN, it was beneficial to promote the formation of the vortex.
3. The surface velocity fluctuation was larger at the 1/4 mold width, and it was smaller at the low-speed zone near the narrow face and the SEN. The level fluctuation increased with an increase in the velocity fluctuation, and the velocity fluctuation increased with the increase in velocity magnitude. The level fluctuation as a function of the surface velocity magnitude was predicted. The results of this study are expected to help us develop and preliminarily verify various ideas for suppress the slag entrainment.

## 6. Future Work

In this study, we primarily discussed the effects of casting speed on the top surface flow characteristics for a 1500 mm × 200 mm mold in a steel plant under constant submergence depth conditions. However, in the actual production, the submergence depth of the SEN was subject to change, especially in the case of the sequence casting. Therefore, it was essential to investigate the effects of different submergence depths on the top surface flow. This not only serves as a supplement to our study but also provides vital evidence for further optimizing the continuous casting process parameters.

Moreover, the effects of superheat on the top surface flow of the mold was not considered in this research. However, in the practical continuous casting production, the superheat of the tundish varied by approximately 10~20 °C. Therefore, exploring the effects of the thermal convection caused by changes in tundish temperature on the flow of the top surface of the steel slab mold was of great significance and value.

## Figures and Tables

**Table 1:** Investigations on the level fluctuation of the top surface in the CC mold.

Year	Authors	Causes of Level Fluctuation on Top Surface	Ref.
1988	Teshima et al.	Impact of molten steel flow on the top surface of the mold	[4]
1991	Herbertson et al.	Bias flow of molten steel in the mold	[20]
1994	Thomas et al.	Disturbance of argon blowing on top surface of the mold	[21]
1994	Codur et al.	Nozzle blockage, stopper rod erosion, and static pressure change in molten steel in the tundish; transient variations in casting speed and slab bulging	[22]
2002	Yoon et al.	Influence of slab bulging under unsteady casting conditions	[23]
2008	Zhang et al.	Serious SEN clogging causing severe level fluctuation of the top surface	[24]
2013	Li et al.	Slab bulging as the root cause of periodic fluctuation of the top surface	[19]
2014	Liu et al.	Disturbance of the stopper rod	[25]
2017	Zhao et al.	Up and down fluctuation of the molten steel jet causing the asymmetric distribution of the flow field and generation of vortices	[26]
2018	Zhu et al.	Large-scale vortex structure in the lower recirculation zone of the mold	[27]
2019	Cho et al.	When the flow field of the mold was a single roll flow pattern, the drastic change in the jet led to a large fluctuation on the top surface.	[28]
2019	Chen et al.	Up and down swing of the molten steel stream from the SEN	[29]
2020	Jiang et al.	Abnormal mold level fluctuation was mainly induced by the shell bulging while moving through the rollers.	[10]
2023	Lei et al.	Abnormal mold level fluctuation of HP steel came from the resonance phenomenon.	[30]

**Table 2:** Casting parameters in the numerical simulation.

Parameter	Value
Mold width × thickness	1500 mm × 200 mm
Mold height	900 mm
Length of calculation domain	3000 mm
Mold work level	800 mm
Inner diameter of SEN	58 mm
Length of SEN	890 mm
SEN outport angle	15° down
Height of SEN port	55 mm
Width of SEN port	45 mm
Submergence depth of SEN	130 mm
Casting speed	0.8 to 1.0 m/min
Thickness of liquid slag	30 mm

**Table 3:** Chemical composition of steel (wt%).

C	Si	Mn	P	S	N	Als	Fe
0.16	0.31	1.35	0.016	0.005	0.003	0.030	98.126

**Table 4:** Chemical composition of original mold flux (wt%).

SiO_2_	CaO	Al_2_O_3_	Fe_2_O_3_	MgO	Na_2_O	K_2_O	MnO_2_	LiO_2_	C
39.94	34.87	7.60	2.14	3.84	1.83	0.47	5.03	1.22	3.06

## Data Availability

Not applicable.
